# Novel patterns of physical activity in a large sample of preschool-aged children

**DOI:** 10.1186/s12889-018-5135-0

**Published:** 2018-02-13

**Authors:** Rachel M. Ruiz, Evan C. Sommer, Dustin Tracy, Jorge A. Banda, Christina D. Economos, Megan M. JaKa, Kelly R. Evenson, Maciej S. Buchowski, Shari L. Barkin

**Affiliations:** 1 Division of Pediatric Gastroenterology, Hepatology, and Nutrition, Stanford University School of Medicine, 750 Welch Road, Suite 116, Palo Alto, CA 94304 USA; 2 Department of Pediatrics, Vanderbilt University Medical Center, 2146 Belcourt Ave, Nashville, TN 37232-9225 USA; 3 Department of Economics, Andrew Young School of Policy Studies, Georgia State University, 14 Marietta St, Atlanta, GA 30303 USA; 4 Stanford Prevention Research Center, Stanford University School of Medicine, Medical School Office Building, 1265 Welch Road, Room X1C39, Stanford, CA 94305-5415 USA; 5 Friedman School of Nutrition Science and Policy, Tufts University, Jaharis Family Center for Biomedical and Nutrition Sciences, 150 Harrison Ave, Boston, MA 02111 USA; 6 Division of Epidemiology and Community Health, School of Public Health, University of Minnesota, 1300 S 2nd St., Suite 300, Minneapolis, MN 55454-1015 USA; 7 Department of Epidemiology, The University of North Carolina at Chapel Hill Gillings School of Global Public Health, 137 East Franklin Street, Suite 306, CVS Plaza, CB #8050, Chapel Hill, NC 27514 USA; 8 Division of Gastroenterology, Hepatology, & Nutrition, Vanderbilt University Medical Center, 2215 Garland Ave, A4103 MCN, Nashville, TN 37232-5280 USA; 9 Department of Pediatrics, Vanderbilt University School of Medicine, 2200 Children’s Way, Doctor’s Office Tower 8232, Nashville, TN 37232-9225 USA

**Keywords:** Physical activity, Childhood obesity, Prevention, Family, MVPA, Latino, Parent and child

## Abstract

**Background:**

Moderate-to-vigorous physical activity (MVPA), shown to be associated with health benefits, is not well-characterized in preschool-aged children. MVPA is commonly described as a threshold amount to achieve. We examined a novel way to characterize MVPA patterns in preschool-aged children by gender and age.

**Methods:**

Preschool-aged children from Nashville, TN and Minneapolis, MN wore triaxial accelerometers. Four distinct MVPA patterns were identified: isolated spurt (IS), isolated sustained activity (ISA), clustered spurt (CS), and clustered sustained activity (CSA). Multivariable linear regression models were used to test associations of gender and age with each pattern.

**Results:**

One thousand one hundred thirty-one children (3.9 years old, 51% girls, 30% overweight, 11% obese, and 76% Hispanic) wore accelerometers for 12.9 (*SD* = 1.4) hours/day for 6.7 (*SD* = 0.7) days. Children spent 53% of wear time in sedentary behavior and 13% in MVPA. On average, boys and girls achieved > 90 min/day of MVPA (98.2 min, *SD* = 32.3). Most MVPA (80%) was obtained in spurt-like (IS and CS) MVPA; however, girls spent a higher proportion of MVPA in IS and CS, and lower proportion of time in CSA (all *p* < 0.001). Controlling for gender, an increase of 1-year in age corresponded to a 1.5% increase in CSA (*p <* 0.05*).*

**Conclusions:**

How MVPA was obtained varied depending on the gender and age of the child. On average, boys spent more time in sustained MVPA than girls and MVPA was more sustained in older children. Utilizing these patterns could inform PA practice and policy guidelines.

**Trial registration:**

NCT01316653, date of registration: March 3, 2011; NCT01606891, date of registration: May 23, 2012.

## Background

The National Academy of Medicine recommends that toddlers and preschool-aged children be provided with opportunities for light, moderate, and vigorous PA for at least 15 min/h [1]. This is equivalent to at least 3 h/day of total PA, similar to international guidelines [2–4]. Nationally representative PA data are not currently available for preschool-aged children; however, a recent US study provides evidence that nearly 60% of preschool-aged children do not meet this guideline [5]. Increasing physical activity (PA), specifically moderate to vigorous PA (MVPA), is important for the prevention and treatment of childhood obesity, health promotion, and beneficial behavioral and academic outcomes [6–10]. This is particularly true for low-income racial/ethnic minority youth. Research has shown that these children have lower odds of meeting PA recommendations [11, 12] and higher odds of being overweight or obese [13, 14]. However, to make specific recommendations on how young children should achieve recommended PA, important data gaps must be addressed. These include understanding MVPA patterns young children engage in rather than simply minutes of MVPA they accumulate, how MVPA patterns evolve as children age, and whether MVPA patterns differ by influencing variables such as gender. Gaining a better understanding of young children’s MVPA behavior has the potential to inform intervention strategies and policies to promote MVPA.

Researchers have begun to explore child PA patterns using accelerometry. PA patterns have been characterized by temporality, such as exploring hourly variability throughout a day or on weekdays compared to weekend days [15–17]. Other studies have defined MVPA patterns by bouts or consecutive minutes above a certain threshold [18–20]. Still others have described PA patterns as the types of activities children are doing, the settings in which these activities are done [21, 22], or by using latent class analysis to define patterns [23, 24]. Most studies focused on older school-aged children. Of the studies that have examined PA patterns in preschool-aged children, most had sample sizes of less than 500 [25]. National data from NHANES demonstrate that boys are more physically active than girls as early as elementary school (6–11 years), and this gender disparity persists into adolescence [26]. Age variations in these patterns have not been studied consistently.

In our prior study of 50 preschool-aged children, we identified 4 specific patterns of MVPA using accelerometry: isolated spurt (IS; 15–60 s bouts of PA, with > 60 s of inactivity preceding and following the MVPA burst), clustered spurt (CS; multiple 15–60 s bouts of PA with ≤60 s between each), isolated sustained activity (ISA; > 60 s bout of PA, with > 60 s of inactivity preceding and following the MVPA burst), and clustered sustained activity (CSA; multiple > 60 s bouts of PA with ≤60 s between each) [27]. Generalizability of these findings were limited by the small sample size and mostly reflected a convenience sample of largely African-American preschoolers. Thus the present study was designed to characterize MVPA patterns in an ethnically diverse large cohort of 2 to 5 year-old children from low-income families and determine whether these patterns differed by gender and age.

## Methods

### Study population and design

Participants were from two obesity prevention treatment trials with preschool-aged children participating in the Childhood Obesity Prevention and Treatment Research (COPTR) Consortium [28, 29]. A total of 1144 children were enrolled in the Vanderbilt University School of Medicine (*n* = 610) and the University of Minnesota (*n* = 534) trials. Inclusion criteria included age between 2 and 5 years, healthy, received some type of federal assistance (e.g., WIC), and a BMI percentile ≥50th. BMI and BMI percentile were calculated using the Center for Disease Control Calculator [30]. The exclusion of children with a BMI <50th percentile allowed inclusion of child participants at-risk for developing obesity. Children were weighed and measured using standard procedures to ensure they met BMI eligibility criterion (≥50th and <95th in the Tennessee cohort, and ≥50th in the Minnesota cohort) [31, 32]. The study was approved by the Vanderbilt University and University of Minnesota, Twin Cities Institutional Review Boards. The data for this study derive from baseline data prior to randomization. Further study design can be found in previous COPTR Consortium manuscripts [31, 32].

### Physical activity measurement and analysis

Participants were instructed to wear an ActiGraph GT3X/GT3X+ accelerometer (ActiGraph, Pensacola, FL) on their right hip for 7 days, including when sleeping and napping. Accelerometry recordings from 7:00 am through 8:59 pm were used to capture the typical daily preschool-aged child wake-time cycle. Adherent wear time criterion was 4–7 days (≥3 weekdays and ≥1 weekend day and a maximum of 7 total days per protocol) with at least ≥360 min of wear time/day.

Raw accelerometry data sampled at 40 Hz were integrated into 15-s and 60-s epochs for PA intensity categories and assessing wear/non-wear intervals, respectively. Non-wear was defined by an interval of at least 90 consecutive minutes of zero counts/min, with allowance of up to 2 min of non-zero counts if no counts were detected during both the 30 min upstream and downstream from that interval; any non-zero counts were considered wear time [33]. PA intensity categories were derived using cutpoints for preschool-aged children (< 38 for sedentary, ≥ 38–419 for light, 420–841 for moderate, ≥ 842 for vigorous, and ≥420 for MVPA all in counts/15-s) [34]. Because we were interested in how combined MVPA was distributed across typical wake-wear hours, only days with ≥360 min of wear between 7:00 am and 8:59 pm were included in analyses. Actigraphy data were processed using the R statistical package [35]. For each occurrence of MVPA, the number of epochs of continuous MVPA and the number of epochs between instances of MVPA were calculated. Based on these, MVPA was classified into one of four MVPA pattern categories [27]:

1. Isolated Spurt (IS): A **single** MVPA period **≤4** epochs (1 min) in length with > 4 epochs of non-MVPA before and after it
2. Isolated Sustained Activity (ISA): A **single** MVPA period **> 4** epochs (1 min) in length with > 4 epochs of non-MVPA before and after it
3. Clustered Spurt (CS): An event comprised of a **series** of MVPA periods that average **≤ 4** epochs (1 min), where there are no periods ≥4 epochs of non-MVPA between periods
4. Clustered Sustained Activity (CSA): An event comprised of a **series** of MVPA periods that average **> 4** epochs (1 min), where there are no periods ≥4 epochs of non-MVPA in between periods.

### Statistical analysis

Univariate statistics were used to describe sociodemographic variables, anthropometric measures, and variables related to accelerometry among children who met minimum accelerometry criteria (*n* = 1131, *n* = 599 Vanderbilt and *n* = 532 Minnesota). Bivariate relationships between child age and each of the 4 MVPA patterns were assessed using nonparametric Spearman’s correlations. Nonparametric, two-sample, Wilcoxon-Mann-Whitney rank-sum tests were conducted to compare distributional differences between boys and girls on variables that demonstrated non-normality. We conducted *t*-tests of the equality of means for two samples to compare boys and girls on normally distributed variables.

To explore if combining data across the 2 trials was appropriate, the interaction effects of study location with child age and gender were both tested for each of the 4 MVPA patterns. Neither of these interactions were statistically significant for any MVPA pattern (*p*-values ranged from 0.09 to 0.90), with the exception of the age-location interaction effect for isolated spurt (*B* = 1.25, *p* = 0.006). We concluded that a pooled analysis was appropriate except for the analysis of the relationship between child age and proportion of time in isolated spurt, for which the results are reported separately by study location.

Multivariable linear regression models were used to test the associations of gender and age with percentage of time in each MVPA pattern. Additional regression models were explored to examine the potential relationships of BMI percentile, study location, and use of child care with each MVPA pattern. In these models, study location was represented as a binary indicator variable that accounted for differences between each study location, such as the different BMI percentile eligibility requirements (Vanderbilt ≥50th to <95th BMI percentile, Minnesota ≥50th BMI percentile). Child care was a binary variable indicating whether the index child participated in child care. Child care type, frequency, and duration were not clarified for all study participants. All calculations and analyses were conducted using Stata/SE 14.1.

## Results

Of the 1144 study participants, 1131 met minimum accelerometry criteria and were included in this study. These children wore accelerometers for averages of 6.7 (*SD* = 0.7) days and 12.9 (*SD* = 1.4) hours/day between 7:00 am and 8:59 pm (Table 1).

**Table 1:** Accelerometer measured average time (min) and % spent in PA intensity categories

	All participants (*n* = 1131)	Girls (*n* = 583)	Boys (*n* = 548)	Test	*P* value^1^	95% CI for difference between boys and girls	
Adherent accelerometer wear days (day)	6.7 (0.7)	6.6 (0.7)	6.7 (0.6)	Rank-sum	0.01	NA	NA
Daily wear time (h)	12.9 (1.4)	12.8 (1.5)	12.9 (1.3)	Rank-sum	0.7	NA	NA
Sedentary							
Mean min (sd)	409.9 (69.6)	413.0 (70.6)	406.5 (68.4)	T-test	0.1	− 14.6	1.7
% wear time	53.2 (7.3)	53.7 (7.3)	52.7 (7.3)	T-test	0.02	−1.83	−0.13
Light							
Mean min (sd)	263.4 (49.0)	265.9 (49.5)	260.8 (48.4)	Rank-sum	0.048	NA	NA
% wear time	34.1 (4.9)	34.4 (4.8)	33.7 (4.9)	Rank-sum	0.008	NA	NA
Moderate							
Mean min (sd)	70.6 (20.6)	67.6 (20.0)	73.8 (20.7)	T-test	< 0.001^2^	3.9	8.6
% wear time	9.2 (2.5)	8.8 (2.4)	9.6 (2.5)	T-test	< 0.001	0.51	1.08
Vigorous							
Mean min (sd)	27.6 (13.7)	24.2 (11.3)	31.3 (14.9)	Rank-sum	< 0.001	NA	NA
% wear time	3.6 (1.7)	3.1 (1.4)	4.1 (1.9)	Rank-sum	< 0.001	NA	NA
Moderate + vigorous							
Mean min (sd)	98.2 (32.3)	91.7 (29.8)	105.2 (33.4)	Rank-sum	< 0.001	NA	NA
% wear time	12.7 (3.9)	11.9 (3.6)	13.6 (4.1)	Rank-sum	< 0.001	NA	NA

Values are means (SD)

^1^*P* values from non-normally distributed variables are from non-parametric, two-sample, Wilcoxon-Mann-Whitney rank-sum tests of the null hypothesis that the distribution of girls is equal to the distribution of boys. *P* values from normally distributed variables are from two-sample *t*-tests of the equality of means

^2^Results from rank-sum and *t*-test agreed. T-test results are reported

### Demographic and anthropometric characteristics

Among participating children about half (48.4%) were males. Mean age was 3.9 (minimum = 2, maximum = 6; *SD* = 0.9) years and mean BMI was 17.1 kg/m^2^ (*SD* = 1.4). The study population was comprised of Hispanic/Latino (75.6%), followed by Non-Hispanic Blacks (11.8%), and Non-Hispanic Whites (6.5%). Nearly one-third (29.9%) of participants were overweight (≥85th to <95th BMI percentile) and 11.5% were obese (≥95th BMI percentile). About 50% of primary caregivers had less than a high school education. Nearly 60% of children lived in a household with an annual household income less than $25,000. Almost 15% of families did not know or declined to report household income. About 30% of children attended child care.

### Physical activity

#### PA intensity

Boys and girls spent about 50% of their wear time in sedentary behaviors. Statistically significant differences between boys and girls were found in time spent in sedentary behavior and all PA activity intensities (light, moderate, vigorous, and MVPA) (Table 1). On average both boys and girls achieved at least 90 min of MVPA/day. However, boys spent a greater percentage of wear time in MVPA than girls (13.6 *SD* = 4.1% and 11.9 *SD* = 3.6%, *p* < 0.001), which translated into 13.5 more minutes of MVPA per day (105.2 *SD* = 33.4 min and 91.7 *SD* = 29.8 min, *p* < 0.001) (Table 1).

#### MVPA patterns

**Fig. 1:**
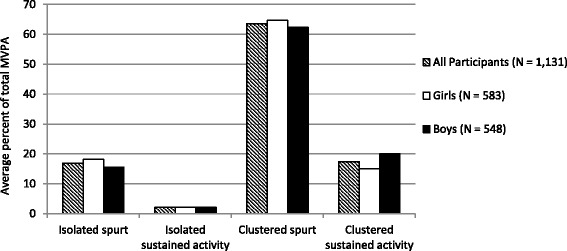
Average percent of total MVPA achieved through each specific pattern type (*N* = 1131). Because the distributions of all PA categories were non-normal, the following *p*-values are from non-parametric, two-sample, Wilcoxon-Mann-Whitney rank-sum tests of the null hypothesis that the distribution of girls is equal to the distribution of boys: IS *p* < 0.001, ISA *p* = 0.2, CS *p* < 0.001, CSA *p* < 0.001

On average, children engaged in isolated spurt 44.0, isolated sustained activity 1.1, clustered spurt 36.5, and clustered sustained activity 2.6 times/day. There were also small differences between boys and girls in the average number of isolated spurt, isolated sustained activity, and clustered sustained activity events per a typical day (Table 2). When the duration of each MVPA pattern event was examined, differences between boys and girls became more pronounced. Boys engaged in each of the four MVPA patterns for longer when compared to girls (all *p* < 0.005) (Table 2). Figure 1 illustrates the percent of MVPA/day achieved in MVPA patterns on an average day.

**Table 2:** MVPA events/day, duration, and time spent in MVPA within each MVPA pattern event (*N* = 1131)

MVPA Pattern	All Participants (*N* = 1131)	Girls (*N* = 583)	Boys (*N* = 548)	Test	*P* value^1^
Isolated spurt					
Frequency (SD)	44.0 (9.1)	44.9 (9.1)	43.1 (8.9)	Rank-sum	< 0.001
Duration (SD)	0.34 (0.02)	0.34 (0.02)	0.34 (0.02)	Rank-sum	0.004*
Isolated sustained activity					
Frequency (SD)	1.1 (0.6)	1.1 (0.5)	1.2 (0.6)	Rank-sum	< 0.001
Duration (SD)	1.73 (0.44)	1.69 (0.42)	1.76 (0.45)	Rank-sum	0.001*
Clustered spurt					
Frequency (SD)	36.5 (9.6)	36.3 (9.8)	36.7 (9.3)	T-test	0.4
Duration (SD)	3.33 (0.62)	3.15 (0.54)	3.52 (0.64)	Rank-sum	< 0.001*
*MVPA duration within clustered spurt event*	2.02 (0.46)	1.89 (0.39)	2.17 (0.48)	Rank-sum	< 0.001*
Clustered sustained activity					
Frequency (SD)	2.6 (1.4)	2.2 (1.2)	3.0 (1.6)	Rank-sum	< 0.001
Duration (SD)	8.35 (2.86)	7.86 (2.76)	8.88 (2.87)	Rank-sum	< 0.001*
*MVPA duration within clustered sustained activity event*	6.69 (2.29)	6.28 (2.18)	7.12 (2.33)	Rank-sum	< 0.001*

*Represents statistically significant *P* values that are  < 0.05

Values are mean (SD); Moderate-to-vigorous physical activity (MVPA)

^1^*P* values from non-normally distributed variables are from non-parametric, two-sample, Wilcoxon-Mann-Whitney rank-sum tests of the null hypothesis that the distribution of girls is equal to the distribution of boys. *P* values from normally distributed variables are from two-sample *t*-tests of the equality of means

##### Gender differences

Compared to boys, girls spent significantly higher proportion of MVPA in isolated spurt (18.2% *SD* = 5.6% vs. 15.5% *SD* = 5.7%) and clustered spurt (64.8% *SD* = 6.7% vs. 62.3% *SD* = 8.1%) (Fig. 1). Boys had a significantly higher proportion of clustered sustained activity compared to girls (20.0% *SD* = 9.5% vs. 15.0% *SD* = 7.9%). All *p*-values were less than 0.001.

##### Age

There was a significant correlation between child age and proportion of time spent in the four MVPA patterns. Older children tended to have a greater proportion of MVPA in clustered sustained activity (*r*_s_ = 0.15, *p* < 0.001) and isolated sustained activity (*r*_s_ = 0.10, *p* < 0.001) and a smaller proportion in clustered spurt (*r*_s_ = − 0.15, *p* < 0.001) and isolated spurt (*r*_s_ = − 0.07, *p* = 0.02).

##### Gender and age

Controlling for age, girls spent an average of 2.4% more MVPA time in clustered spurt than boys (*p* < 0.001*,* 95% CI for mean difference 1.52, 3.24). Controlling for gender, a 1-year increase in age corresponded to a 1.34% decrease (*p* < 0.001; 95% CI for mean difference 0.88, 1.81) in the proportion of MVPA time spent in clustered spurt (Table 3).

**Table 3:** Linear associations of child gender and age with time spent in each PA pattern category

PA pattern category (MVPA %)	Gender coefficient^a^	Gender *p* value	95% CI		Age coefficient	Age *p* value	95% CI	
Isolated spurt % (MN)^b^	3.02	< 0.001	2.09	3.96	−1.32	< 0.001*	−2.03	−0.60
Isolated spurt % (VU)^b^	2.44	< 0.001	1.51	3.36	−0.07	0.8	−0.59	0.45
Isolated sustained activity % (pooled)	−0.11	0.1	−0.25	0.04	0.18	< 0.001*	0.10	0.26
Clustered spurt % (pooled)	2.38	< 0.001	1.52	3.24	−1.34	< 0.001*	− 1.81	−0.88
Clustered sustained activity % (pooled)	−5.01	< 0.001	−6.02	−4.01	1.54	< 0.001*	1.00	2.09

*Represents statistically significant *P* values that are  < 0.05

^a^Represents estimated change from male to female; Moderate-to-vigorous physical activity (MVPA)

^b^Isolated spurt results are reported separately by study location because of a significant study location-by-age interaction effect (*B* = 1.25, *p* = 0.006)

Compared to boys, girls spent an average of 2.4 (VU)-3.0 (MN)% more MVPA time in isolated spurt than boys (*p* < 0.001*;* 95% CI 2.09, 3.96 (VU) versus 1.51, 3.6 (MN)). A 1-year increase in age corresponded to a 1.3% decrease in the proportion of MVPA time in isolated spurt at MN, but no relationship was detected at VU. Boys spent on average 5.0% more MVPA time in clustered sustained activity than girls (*p* < 0.001*;* 95% CI for mean difference 4.01, 6.02). A 1-year increase in age corresponded to a 1.5% increase in the proportion of MVPA time spent in clustered sustained activity (*p* < 0.001; 95% CI for mean difference 1.00, 2.09).

##### BMI percentile, study location, and child care

Regression models identical to those explored for gender and age were also explored with the addition of covariates for BMI, study location, and child care. None of these covariates were statistically significant, nor did their inclusion change the interpretations of the models.

## Discussion

The aim of this study was to assess the prevalence and frequency of novel MVPA patterns (isolated spurt, clustered spurt, isolated sustained activity, clustered sustained activity) in a large sample of typically developing preschool-aged children from socioeconomically disadvantaged families at-risk for obesity. In our study, the majority of children achieved the National Academy of Medicine recommendation of at least 3 h/day of total PA including at least 90 min of MVPA, but how they obtained it varied depending on the gender and age of the child. Boys obtained ~ 13.5 more minutes of MVPA per day than girls, which translated to an additional 94.5 min/week. This seemingly small daily difference could have differential health impacts and should be further assessed. Boys and girls also differed in the relative amount of time they spent in each MVPA pattern. While girls spent a statistically significant greater proportion of their MVPA time in spurt-like MVPA patterns, boys spent a statistically significant greater proportion of their MVPA time in sustained MVPA patterns.

In particular, boys and girls differed in the average duration of a single MVPA pattern, specifically in the clustered spurt and clustered sustained activity categories. A clustered spurt event for boys averaged an additional 22 s in duration (17 of which were MVPA) when compared to girls. This seemingly small time difference, when accumulated throughout the day, translated to about 10 more minutes of MVPA for boys from this MVPA pattern alone. Additionally, a clustered sustained activity event for boys averaged an additional 61 s in duration (50 of which were MVPA) when compared to girls; this translates to an additional 7 min of MVPA for boys on average. These findings are promising since research among preschool-aged children have found an inverse relationship between physical activity and BMI [36]. Furthermore, children that demonstrated high-intensity physical activity were less likely to be overweight and exhibit body fatness during the adiposity rebound period of childhood growth [37, 38]. Additionally, these findings could give us insight into the gender gap that emerges in older children [39].

PA is associated with a number of health benefits in youth, regardless of their BMI status [40]. The literature provides support for a relationship between greater PA and favorable health outcomes such as adiposity, bone and skeletal health, psychosocial health, cognitive development, and cardiometabolic health among children 4-years and younger [41]. Literature remains largely inconclusive regarding whether a significant difference exists in the daily amount of MVPA between preschool-aged boys and girls [16, 27, 42–44]. In our study, we did find significant differences of about 6.2 min/day of moderate PA and 7.1 min/day of vigorous PA between boys and girls. Girls were active, but spent less time in MVPA and less time in *sustained patterns* of MVPA (isolated sustained activity, clustered sustained activity). The importance of studying MVPA in preschool-aged children extends beyond establishing whether or not the recommended daily level is achieved. Examining PA through these novel MVPA patterns may contribute to our understanding of the inherent differences between boys and girls.

It has been estimated that about 61% of children under 5 years of age in the US attend some form of child care regularly during a typical week [45]. Child care arrangements have been shown to vary greatly in the amount of MVPA children obtain during their care [46, 47]. Among our study cohort, no difference in percentage of time in any MVPA pattern was detected between children who attended child care and those who did not. More research is needed to examine whether the structure of daycare time allotted to PA can enhance time spent in MVPA.

In one particular study that looked at outdoor free time among preschoolers, children were noted to be more active in the first 10–15 min of playtime compared to the last 10–15 min of playtime. The 3 year-old participants had a more significant difference between first and last minutes of playtime compared to their 4 and 5 year-old counterparts [48]. Our current study found that among preschoolers, each additional year of age was associated with less time in spurt-like activity and more time in sustained activity. This finding aligns with our a priori hypothesis about the relationship between age and sustained and spurt-like activity, and it also supports the suggestion that older preschool-aged children are active for longer periods of time compared to younger preschool-aged children. If we take these results to illustrate the child development and associated PA behaviors of preschool-aged children, it follows that short duration PA activities (< 15 min) offered at several times throughout the day might help children maximize their MVPA. The novel findings reported in this study could allow us to examine new hypotheses to influence PA practices and policies for young children.

### Limitations

Despite our extremely compliant large cohort of preschool-aged children who provided us with a robust dataset for analysis, some limitations exist. By study design, we did not examine PA in any child under the 50th BMI percentile. Our study cohort was predominately comprised of Hispanic/Latino children from low SES households. Thus, we cannot generalize our data to children from higher socioeconomic status, who are white, or whose BMI percentile is <50th. In our study, a participant’s neighborhood environment was not controlled for in our statistical analysis, and thus could be a potential confounder. Additionally, we did not have the ability to distinguish sedentary behaviors from sleep, so we could not make any conclusions about sedentary behavior; however, that was not the intention of this study. Lastly, this was a cross-sectional study; thus, we could only look at correlations not causation.

## Conclusions

Most underserved minority preschool-aged children in this cohort obtained the current daily recommended amount of MVPA during their waking day. However boys and girls achieved this recommendation through different patterns of MVPA, girls through more spurt-like PA and boys through more sustained PA. Within the preschool-aged population, each additional year in age was associated with less time spent in spurt-like patterns of PA and more time spent in sustained patterns of PA. If these patterns persist as children age, these differences could, in part, explain the gender gap in MVPA.

## Abbreviations

BMI: Body Mass Index
COPTR: Childhood Obesity Prevention and Treatment Research
CS: Clustered Spurt
CSA: Clustered Sustained Activity
IS: Isolated Spurt
ISA: Isolated Sustained Activity
MVPA: Moderate to Vigorous Physical Activity
PA: Physical activity
